# Carbonated drinks, chips intake and their relation to Intelligence Quotient (IQ) among primary school children in Baghdad city, Iraq

**DOI:** 10.1186/1471-2458-12-S2-A19

**Published:** 2012-11-27

**Authors:** Hasanain Faisal Ghazi, Zaleha Md Isa, Mohammed A AbdalQader, Isidore Koffi Kouadio, Azam Rahimi, Nimetcan Mehmet Orhun, Syed Mohamed Aljunid

**Affiliations:** 1 Department of Community Health, Universiti Kebangsaan Malaysia Medical Centre, Jalan Yaacob Latiff, 56000 Kuala Lumpur, Malaysia; 2 United Nations University- International Institute for Global Health, Universiti Kebangsaan Malaysia Medical Centre, Jalan Yaacob Latiff, 56000 Kuala Lumpur, Malaysia

## Background

The revolution in children’s lifestyle and dietary habits which has occurred over the last thirty years can be largely attributed to changes in the family environment and in the social environment in general. Fast food is a diet high in processed foods and soft drinks. Children consuming more junk food are likely to have a lower intake of vitamins, minerals and essential fatty acids. The aim of this study was to assess the relationship between carbonated drinks, chips intake and intelligence quotient of primary school children.

## Methods

A cross sectional study was conducted in Baghdad city, Iraq. A self-administered questionnaire was distributed to children’s parents to elicit information on children food habits at school. Raven colored progressive matrices were used to obtain child’s IQ score, any score more than 75th percentile was considered as high intelligence level.

## Results

A total of 529 children participated in this study, (77.7%) of the children have high IQ level. Almost (60%) of the children drink carbonated drink daily, while (74%) eat chips every day. The association between drinking carbonated drinks, eating chips and intelligence level was significant (P= 0.043, 0.001) and prevalence odds ratio of 1.5 and 2.4 respectively.

## Conclusions

There were significant associations between carbonated drinks, chips intake and IQ score. More regulations on what to sell inside the schools canteen are needed. Increasing nutritional knowledge of the parents, especially the mothers is very important and more health promotions should be given regarding children nutrition in the early years of school.

